# Editorial: Protein homeostasis in host-pathogen interactions

**DOI:** 10.3389/fmicb.2022.1115857

**Published:** 2023-01-04

**Authors:** Jinki Yeom, Donghyuk Shin, Yuan Qiao

**Affiliations:** ^1^Department of Biomedical Sciences, College of Medicine, Seoul National University, Seoul, Republic of Korea; ^2^Department of Microbiology and Immunology, College of Medicine, Seoul National University, Seoul, Republic of Korea; ^3^Cancer Research Institute, Seoul National University, Seoul, Republic of Korea; ^4^Department of System Biology, College of Life Sciences and Biotechnology, Yonsei University, Seoul, Republic of Korea; ^5^Institute of Molecular and Cell Biology, Agency for Science, Technology and Research, Singapore, Singapore; ^6^School of Physical and Mathematical Sciences, Nanyang Technological University, Singapore, Singapore

**Keywords:** infection, protein homeostasis, host-pathogen interaction, immune response, pathogen

All living organisms maintain protein homeostasis, which regulates survival and proliferation (Hoppe and Cohen, 2020). Stress conditions can disrupt protein homeostasis by impacting translation, protein folding, and proteolysis (Arnsburg and Kirstein-Miles, 2014). Pathogens encounter stress conditions during infection, such as nutritional starvation and oxidative stress, and, thus, must maintain protein homeostasis to survive in the host environment (Fang et al., 2016). On the other hand, when host cells meet pathogens during infection, they regulate their own proteome to inhibit pathogen growth and to activate an immune response (Denzer et al., 2020). Host and pathogens try to maintain protein homeostasis during infection to defeat each other.

Pathogens encounter harsh environments by the host immune system, which enables a disruption of protein homeostasis. First, macrophage and neutrophil cells try to engulf pathogens, and then destroy the pathogens by autolysosome. To survive and proliferate inside innate immune cells, pathogens need to overcome ROS, RNS, acidic pH, and nutritional starvation (Silva and Correia-Neves, 2012). Second, host cells can sequester essential metals for pathogen growth, such as iron, zinc, and magnesium (Hennigar and McClung, 2016). Third, immunes cells produce antimicrobial peptides to disrupt membrane and cell wall in pathogens (Boparai and Sharma, 2020). Fourth, commensal microbiota can trigger the host immune response, which activates the host defense system against pathogens (Belkaid and Hand, 2014). In summary, pathogens need to overcome stress conditions from host environments during infection, which can disturb protein homeostasis in pathogens.

There are several examples of how microorganisms control the host immune response by regulating protein homeostasis. First, Horianopoulos et al. determined that the fungal pathogen *Cryptococcus neoformans* produces Dnj1 chaperone by sensing elevated temperatures during infection. Dnj1 is required for protein homeostasis by regulating protein folding, compromising the host immune system, and maintaining membrane homeostasis. Chaperone, a key factor for protein homeostasis, governs pathogenesis and survival during fungal pathogen infections. Second, iron is an essential metal for both the host and the pathogen during infection. Chantes-Guerra et al. revealed that the mammalian pathogenic bacterium *Gallibacterum anatis* secrets siderophore, a natural chelator to capture iron from the host, to obtain iron. Receptor proteins in bacteria should recognize a siderophore-iron complex to uptake iron. *G. anatis* regulates proteome to produce additional siderophore receptor systems that maintain iron homeostasis in pathogens. This could benefit pathogenic bacterium to survive and proliferate in the host. Third, gut microbiota is essential to defend against pathogens by regulating host immune response and enhancing production of mucosal barrier. Huang et al. reported that berberine, a natural extract of *Rhizoma Coptidis*, becomes probiotic by promoting butyrate production, which can reduce cancer cell growth in colon cancer. It confers change of microbiota composition by metabolic production, which leads to reduced cancer cell growth.

In host-pathogen interaction, host cells regulate protein homeostasis to control pathogen growth during infection. First, the host rearranges proteolytic pathways during infection (Cornejo et al., 2017). Host cells attach ubiquitin to the pathogens' membrane proteins, which cleans up pathogens by autophagy. Several proteins are highly activated by invading pathogens, thereby increasing ubiquitination and activating an autophagy pathway. Second, metal ions can impact protein homeostasis by regulating protein folding, conformational change, cellular communication, and catalysis. Host cells capture essential metal ions to inhibit pathogen growth, a process that is termed nutritional immunity and that leads to proteome rearrangement (Hennigar and McClung, 2016). Third, host cells activate defense enzymes and carrier proteins for ROS to impede pathogen growth. Innate immune cells produce radicals to kill pathogens from the mitochondria (Silwal et al., 2020). Mitochondrial ROS can generate signaling responses and changes in nuclear gene expression in multiple ways, thus host cells endeavor to maintain protein homeostasis (Silwal et al., 2020). Together, host cells try to maintain protein homeostasis because proteome of host cells is impacted by invading pathogens during infection.

There are several examples of how host cells regulate pathogen growth by protein homeostasis. First, Guo et al. investigated that the steroid hormone estrogen is involved in the immunomodulatory processes of *Neisseria meningitidis* infection. Estrogen binds to estrogen receptor β, regulates the p38-MAPK signaling pathway, and inhibits the release of inflammatory factors during bacterial infection, thus reducing the occurrence and development of the inflammatory response of host cells after *N. meningitidis* infection. Thus, host cells use steroid hormones to maintain cellular homeostasis by regulating immune signaling during infection. Second, dengue virus (DENV) is spread out to people through the bite of an infected *Aedes* species mosquito. Wang et al. established that the mosquito rearranges overall proteome during DEVN infection, which maintains homeostasis of protein and immune response. The mosquito enriches proteins related to the ubiquitin ligase complex, the structural constituent of cuticle, the carbohydrate metabolism, and lipid metabolism pathways, which promotes virus replication in the vector and the transmission to the animal. In summary, host cells modulate proteome to regulate pathogen proliferation.

## Conclusion

Host and pathogens continuously interact, which leads to niche modulating environments during infection. Homeostasis of proteome is critical for host-pathogen interaction because all living organisms regulate biological activities by proteins. Currently, it is poorly understood how host and pathogen maintain protein homeostasis during infection. Thus, more comprehensive and disease-specific studies are necessary to investigate the mechanism of proteome homeostasis during infection.

## Author contributions

JY wrote the draft, with input from DS and YQ. JY revised the final version. All authors listed have made a substantial, direct, and intellectual contribution to the work and approved it for publication.
